# Thoracic and abdominal diagnostic imaging findings in dogs diagnosed with immune‐mediated polyarthritis: 71 cases (2011‐2023)

**DOI:** 10.1111/jsap.13818

**Published:** 2024-12-26

**Authors:** L. Atkinson, F. Schiborra, E. O'Connell, J. Barton, W. Humphreys

**Affiliations:** ^1^ Small Animal Teaching Hospital University of Liverpool Neston UK

## Abstract

**Objectives:**

To describe thoracic and abdominal imaging findings in dogs with immune‐mediated polyarthritis and to evaluate their impact on the decision to commence immunosuppressive therapy.

**Materials and Methods:**

Retrospective case series describing imaging findings in dogs with immune‐mediated polyarthritis across modalities, including thoracic radiographs, abdominal ultrasound, computed tomography, and where available, echocardiography. Additionally, two internal medicine clinicians reviewed the signalment, clinical signs, clinicopathological findings and diagnostic imaging results on two separate occasions, reaching a consensus for each dog on whether immunosuppression would be their treatment of choice or whether their recommendations would be altered by the results of diagnostic imaging.

**Results:**

Seventy‐one dogs met the inclusion criteria. Abnormal diagnostic imaging findings were identified in 25.4% (18/71) of dogs. Thoracic radiography did not identify significant imaging findings in any of the dogs. Lymphadenomegaly was the most commonly reported finding on computed tomography (32/40) and abdominal ultrasound (13/34). Neoplasia was identified in three dogs (3/71). Four (4/13) dogs had echocardiography findings consistent with endocarditis. Immunosuppression without performing diagnostic imaging would have been considered in 41 of 71 (57.7%) dogs, based on the signalment, presenting signs, results of physical examination and clinicopathological testing. Of these, 10 dogs (24.3%) had diagnostic imaging findings suggestive of an underlying trigger, therefore changing the clinician's decision to proceed with immunosuppression.

**Clinical Significance:**

Abdominal imaging and echocardiography should be prioritised over thoracic radiography, in dogs with immune‐mediated polyarthritis. Signalment, presenting complaint, physical examination findings and clinicopathological results are not reliable predictors of abnormal diagnostic imaging findings in dogs with immune‐mediated polyarthritis.

## INTRODUCTION

Immune‐mediated polyarthritis (IMPA) is an inflammatory joint disease which can significantly impact the welfare of affected dogs (Johnson & Mackin, 2012a; Ravicini et al., 2023). In a referral setting, its incidence is reported to be up to 20% among dogs investigated for pyrexia of unknown origin (Battersby et al., 2006; Dunn & Dunn, 1998), while mortality and relapse rates can be as high as 20% and 50%, respectively (Clements et al., 2004; Ravicini et al., 2023). Although most dogs are presented with non‐specific complaints including inappetence, lethargy and pyrexia, many experience significant discomfort and pain, making them reluctant or unable to ambulate (Johnson & Mackin, 2012b; Ravicini et al., 2023; Stull et al., 2008).

Idiopathic IMPA, also referred to as primary or Type I, is reportedly most common (Johnson & Mackin, 2012a; Ravicini et al., 2023; Stull et al., 2008). Erosive and non‐erosive forms of idiopathic IMPA have been described, with the former being characterised by osteolysis of subchondral bone of the affected limbs and poorer prognosis (Johnson & Mackin, 2012a; Shaughnessy et al., 2016; Whitworth et al., 2019). Systemic inflammation or infection (Type II), gastrointestinal disease (Type III) and neoplasia (Type IV) form the most common underlying causes in secondary IMPA (Clements et al., 2004; Johnson & Mackin, 2012a).

Diagnosis of IMPA is based on the presence of sterile neutrophilic inflammation in arthrocentesis samples from multiple joints (Johnson & Mackin, 2012b; Whitley, 2007).

Treatment of primary IMPA often involves non‐specific immunosuppression, with a single or multi‐agent approach (Fukushima et al., 2021; McKee, 2013; Ravicini et al., 2023). However, treatment of secondary IMPA is focused on specific treatment of the underlying disease, for example, endocarditis or vector‐borne diseases. Immunosuppression may result in clinical deterioration in dogs with secondary IMPA. Diagnostic imaging plays an important role in excluding underlying causes (Indzhova et al., 2023; Johnson & Mackin, 2012b; Ravicini et al., 2023; Webb et al., 2002), as well as directing sampling of abnormal tissues for further diagnostics, including cytology and histopathology.

Studies describing imaging findings in dogs with IMPA are limited, predominantly reporting musculoskeletal radiographic abnormalities (McKee, 2013; Shaughnessy et al., 2016). A recently published study discusses thoracic radiographic and abdominal ultrasound findings in a referral population of dogs with IMPA (Tang et al., 2024), utilising a previously established scoring system to assess the utility of these findings (Andres et al., 2019). In this study, the authors conclude that thoracic radiography and abdominal ultrasonography findings are not useful in the overall management of the majority of dogs with IMPA (Tang et al., 2024). However, to date there are no studies describing thoracic and abdominal findings in dogs with IMPA across imaging modalities, nor do the available studies investigate the effect of the signalment, presenting signs, clinical exam and clinicopathological findings on the decision to perform diagnostic imaging in dogs with IMPA.

The purpose of this retrospective single‐institution descriptive study was thus two‐fold: first, to describe multimodality imaging findings in dogs diagnosed with IMPA; second, to characterise the clinical utility of these findings in aiding clinicians to decide which dogs to immunosuppress.

## MATERIALS AND METHODS

Ethical approval was obtained from the University of Liverpool ethical review committee.

Electronic medical (Tristan, Practice Management Software) and imaging records (Microsoft Sharepoint Online, Microsoft‐365) from the University of Liverpool Small Animal Teaching Hospital were searched by the first author in February 2023 for dogs diagnosed with IMPA, from January 2011 to January 2023, using the following key words: immune‐mediated polyarthritis, IMPA, arthrocentesis and joint fluid. To be included, dogs had to have a confirmed diagnosis of IMPA based upon the presence of sterile neutrophilic inflammation in synovial fluid from at least three joints, with a total nucleated cell count of >3000/μL and consisting of >10% neutrophils (Clements et al., 2004; Whitley, 2007). Additionally, all dogs had to have undergone thoracic and abdominal imaging; either radiography and/or ultrasound, or computed tomography (CT) as part of the initial investigations.

Dogs were excluded if treatment was initiated prior to diagnostic imaging, if the arthrocentesis results were inconclusive or if only musculoskeletal imaging was performed.

For all dogs that met the inclusion criteria, the following information was collated from their medical records: breed, age, sex, neuter status, presenting signs, physical examination findings, complete blood count, biochemistry, urinalysis, infectious agent serology and C‐reactive protein concentration.

The imaging included a combination of at least two orthogonal thoracic radiographs and/or abdominal ultrasound or CT of thorax and abdomen. In some cases, echocardiography was also performed.

For radiographic examination, the dogs had at least dorsoventral and right lateral thoracic radiographs, with the addition of the left lateral projection at the discretion of the attending radiologist. All radiographs were obtained at a single institution using two machines – Fujifilm CR prior to 2018, and Fujifilm DX (Fujifilm Healthcare, Bedford, UK) for all studies performed thereafter. Tube potential and mAs were selected according to the standard institutional protocol based on the dog's body mass.

For abdominal ultrasound, all dogs were scanned in lateral recumbency, using a Samsung RS80 EVO (MIS Healthcare, London, UK) and GE Logiq S7 Expert (IMV Imaging, Bellshill, UK). A complete abdominal ultrasound examination was performed for every dog by a European College of Veterinary Diagnostic Imaging (ECVDI) resident under the supervision of an ECVDI specialist.

CT of the thorax and abdomen was performed with a Canon Aquilion Prime 80 slice scanner (Canon Medical Systems Ltd, Crawley, UK), with dogs in sternal recumbency. Images were reconstructed using the standard protocol at the authors' institution. Typical acquisition parameters included 0.5 mm slice thickness, 120KV, automatically modulated tube current, with soft tissue reconstruction of the thorax and the abdomen, and lung reconstruction for the thorax. Non‐ionic iodine‐based contrast (Optiray 300 mg I/ml) was injected using a power injector (Medrad Stellant, Bayer, Reading, UK) at a dose of 2 mL/kg. Bolus tracking was used for triggering the arterial phase at 180HU with a region of interest positioned in the descending aorta.

All imaging studies were performed under sedation or general anaesthesia using a protocol approved by a European College of Veterinary Anaesthesia and Analgesia (ECVAA) resident or specialist based on each dog's ASA grade and expected diagnostic algorithm. A report was composed for each study by an ECVDI resident and verified by an ECVDI specialist at the time of image acquisition.

For the purposes of this study, all images were reviewed retrospectively by two ECVDI radiologists (FS and WH), unblinded to the purpose of the study. Imaging findings were recorded for subsequent analysis.

Imaging findings at the time of image acquisition were used to direct fine needle aspiration of suspected abnormalities by the attending radiologist. All obtained samples were submitted for analysis by a clinical pathologist and the results were reviewed by the author (LA).

Right and left parasternal echocardiography was performed with a GE Vivid E95 (GE Medical Systems Ltd, Buckinghamshire, UK) by European College of Veterinary Internal Medicine Cardiology residents under supervision or specialists, with a complete written report composed at the time of the exam.

To permit assessment of the utility of thoracic and abdominal imaging based on the clinical outcome of immunosuppression, one European College of Veterinary Internal Medicine specialist and one residency‐trained internal medicine clinician were presented with two scenarios. All cases were randomised and anonymised for each scenario. Initially, the clinicians were presented with only the signalment, presenting complaint, physical examination and clinicopathological findings. Subsequently, two weeks later, they were presented with solely the imaging findings. For each scenario, the clinicians were asked to decide in consensus, blinded to the outcome, whether immunosuppression would be the treatment of choice based on the available information. The number of cases where the treatment decision changed based on the imaging findings was recorded by the author (LA).

## RESULTS

### Population

Seventy‐three client‐owned dogs met the initial selection criteria. Two dogs were subsequently excluded. One dog had inconclusive arthrocentesis results due to blood contamination and the original images performed at the time of diagnosis in the other dog could not be located. The remaining 71 dogs were included in the study. The cohort included 29 males, of which 17 were castrated and 42 females, of which 31 were spayed. Thirty‐two breeds were represented, including 12 cross breed, seven English Cocker spaniels, six Labrador retrievers, five whippets, four German shepherd dogs, four English Springer spaniels, three miniature Schnauzers, three Border collies, three Bernese Mountain dogs, two Cavalier King Charles spaniels, two greyhounds, two toy Poodles, two lurchers and one each of: golden retriever, English bulldog, Lhasa apso, great dane, Shetland sheepdog, beagle, Bolognese, Jack Russell terrier, Old English Sheepdog, Hungarian vizsla, West Highland White terrier, boxer, German Wirehaired Pointer, German Spitz, Newfoundland, Staffordshire Bull terrier and Lakeland terrier.

The mean age of the dogs included was 5 years 5 months (range 3 months to 11 years), with a mean weight of 19.8 kg (range 4.3 to 50.6 kg).

### Clinicopathological findings

The most common clinical signs and physical examination findings were: pyrexia 43 of 71 (60.5%), lethargy 34 of 71 (47.8%), lameness 32 of 71 (45%), reluctance or inability to move 21 of 71 (29.5%), hyporexia 17 of 71 (23.9%), peripheral lymphadenomegaly 17 of 71 (23.9%), generalised pain 16 of 71 (22.5%), cardiac murmur eight of 71 (11.2%) weakness six of 71 (8.4%), weight loss five of 71 (7%), ulceration of the tongue, nose and soft palate three of 71 (4%), coughing three of 71 (4%), retching two of 71 (2.8%) and diarrhoea two of 71 (2.8%).

C‐reactive protein concentration was measured in 53 of 71 dogs and was increased in 52 (98.1%) dogs, with a mean value of 98.53 mg/L (reference <10 mg/L). Other common clinicopathological abnormalities included: increased alkaline phosphatase (ALP) activity (24/71; mean 437.33 IU/L; reference 0 to 130 IU/L), hypoalbuminemia (23/71; mean 21.57 mmol/L; reference 26.3 to 38.2 mmol/L), mature neutrophilia (25/71; mean 19.54 10^9^/L; reference: 3 to 12 10^9^/L); and proteinuria (20/71; mean UPCR 2.91 mg/dL; reference <0.2 to 0.5 mg/dL). Vector‐borne disease testing (SNAP 4Dx, IDEXX Laboratories) was performed in 38 of 71 dogs, with zero testing positive.

### Diagnostic imaging

Thoracic radiographs, abdominal ultrasound and CT of the two regions were performed in 31 of 71 (43.6%), 34 of 71 (47.8%) and 40 of 71 (56.3%) dogs, respectively. Thirty‐one dogs had a combination of thoracic radiographs and abdominal ultrasound, two dogs had only abdominal ultrasound and one dog had a combination of abdominal ultrasound and CT performed. Thoracic and abdominal CT was performed in 37 dogs without the addition of another modality, not including sampling under ultrasound guidance. Two dogs had imaging with all three modalities. Echocardiography was performed in 13 of 71 (18.3%) dogs, eight (61.5%) of which had a cardiac murmur.

Thoracic radiographs were reported as normal in 18 dogs (58%), with only non‐specific findings identified in the remaining 13 dogs, including bronchointerstitial (3/13) and interstitial (2/13) pulmonary patterns. All thoracic radiographic findings are listed in Table 1.

**Table 1:** Thoracic imaging (radiography and computed tomography) findings in dogs with IMPA

	Radiographs (*n* = 31), *n* (%)	Computed tomography (*n* = 40), *n* (%)	Total (*n* = 71), *n* (%)
Normal	18 (58)	12 (30)	30 (42)
Lymphadenomegaly	0 (0)	24 (60)	24 (33.8)
Ground glass attenuation	0	6 (15)	6 (8.4)
Bronchointerstitial pattern	3 (9.6)	1 (2.5)	4 (5.6)
Alveolar pattern	1 (3.2)	2 (5)	3 (4.2)
Interstitial pattern	2 (6.4)	1 (2.5)	3 (4.2)
Bronchial wall thickening	2 (6.4)	1 (2.5)	3 (4.2)
Cardiomegaly	2 (6.4)	0 (0)	2 (2.8)
Pulmonary mass/nodule	0 (0)	2 (5)	2 (2.8)
Mediastinal nodule	0 (0)	1 (2.5)	1 (1.4)
Subpleural nodule/thickening	0 (0)	1 (2.5)	1 (1.4)
Subcutaneous mass	0 (0)	1 (2.5)	1 (1.4)
Bronchial wall mineralisation	1 (3.2)	0 (0)	1 (1.4)
Redundant tracheal membrane	1 (3.2)	0 (0)	1 (1.4)
Mineralisation in thoracic wall	1 (3.2)	0 (0)	1 (1.4)
Heart base nodule	0 (0)	1 (2.5)	1 (1.4)

*n* Number of dogs

**FIG 1:**
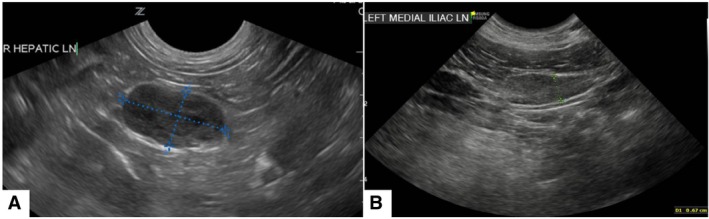
Ultrasound images of neoplastic and reactive lymph nodes. (A) Enlarged hypoechoic right hepatic lymph node with rounded margins: histiocytic sarcoma confirmed on cytology. (B) Enlarged left medial iliac lymph node with normal echogenicity and margination, reactive hyperplasia confirmed on cytology.

Abdominal ultrasound examination was considered normal in six of 34 (17.6%) dogs. The most common ultrasonographic abnormality was lymphadenomegaly 13 of 34 (38.2%) (Fig 1), with the medial iliac lymph node reported as enlarged in 12 of 13 dogs. Additional abnormal ultrasound findings are summarised in Table 2.

**Table 2:** Abdominal imaging (ultrasound and computed tomography) findings in dogs with IMPA

	Ultrasound (*n* = 34), *n* (%)	Computed tomography (*n* = 40), *n* (%)	Total (*n* = 71), *n* (%)
Normal	6 (17.6)	5 (12.5)	11 (15.4)
Lymphadenomegaly	13 (38.2)	23 (57.5)	36 (50.7)
Splenic nodule/mass	6 (17.6)	6 (15)	12 (16.9)
Gallbladder sediment	9 (26.4)	2 (5)	11 (15.4)
Hepatic nodule/mass	4 (11.7)	4 (10)	8 (11.2)
Small adrenal glands	4 (11.7)	4 (10)	8 (11.2)
Renal infarcts	0 (0)	7 (17.5)	7 (9.8)
Hepatomegaly	4 (11.7)	2 (5)	6 (8.4)
Urinary bladder sediment	6 (17.6)	0 (0)	6 (8.4)
Adrenal nodule	3 (8.8)	1 (2.5)	4 (5.6)
Gastric foreign body	1 (2.9)	3 (7.5)	4 (4.6)
Reduction of renal corticomedullary definition	4 (11.7)	0 (0)	4 (4.6)
Adrenomegaly	2 (5.8)	1 (2.5)	3 (4.2)
Splenomegaly	0 (0)	3 (7.5)	3 (4.2)
Uterine fluid accumulation	0 (0)	3 (7.5)	3 (4.2)
Pancreatic enlargement	1 (2.9)	1 (2.5)	2 (2.8)
Common bile duct dilation	0 (0)	2 (5)	2 (2.8)
Small intestinal wall thickening	2 (5.8)	0 (0)	2 (2.8)
Peritoneal effusion	2 (5.8)	0 (0)	2 (2.8)
Prostatomegaly	2 (5.8)	0 (0)	2 (2.8)
Hyperechoic renal cortex	2 (5.8)	0 (0)	2 (2.8)
Heterogenous pancreas	1 (2.9)	0 (0)	1 (1.4)
Pancreatic nodule	1 (2.9)	0 (0)	1 (1.4)
Portal vein thrombosis	0 (0)	1 (2.5)	1 (1.4)
Irregular gall bladder wall lining (adhered sediment vs. mucosal hyperplasia)	1 (2.9)	0 (0)	1 (1.4)
Mammary nodule	0 (0)	1 (2.5)	1 (1.4)
Swollen uterus and mammary tissue	0 (0)	1 (2.5)	1 (1.4)
Generalised loss of pyloric wall layering	1 (2.9)	0 (0)	1 (1.4)
Gastric wall thickening	1 (2.9)	0 (0)	1 (1.4)
Hyperechoic liver parenchyma	1 (2.9)	0 (0)	1 (1.4)
Cystic lesions in prostate	1 (2.9)	0 (0)	1 (1.4)
Pyelectasia	1 (2.9)	0 (0)	1 (1.4)

*n* Number of dogs

**FIG 2:**
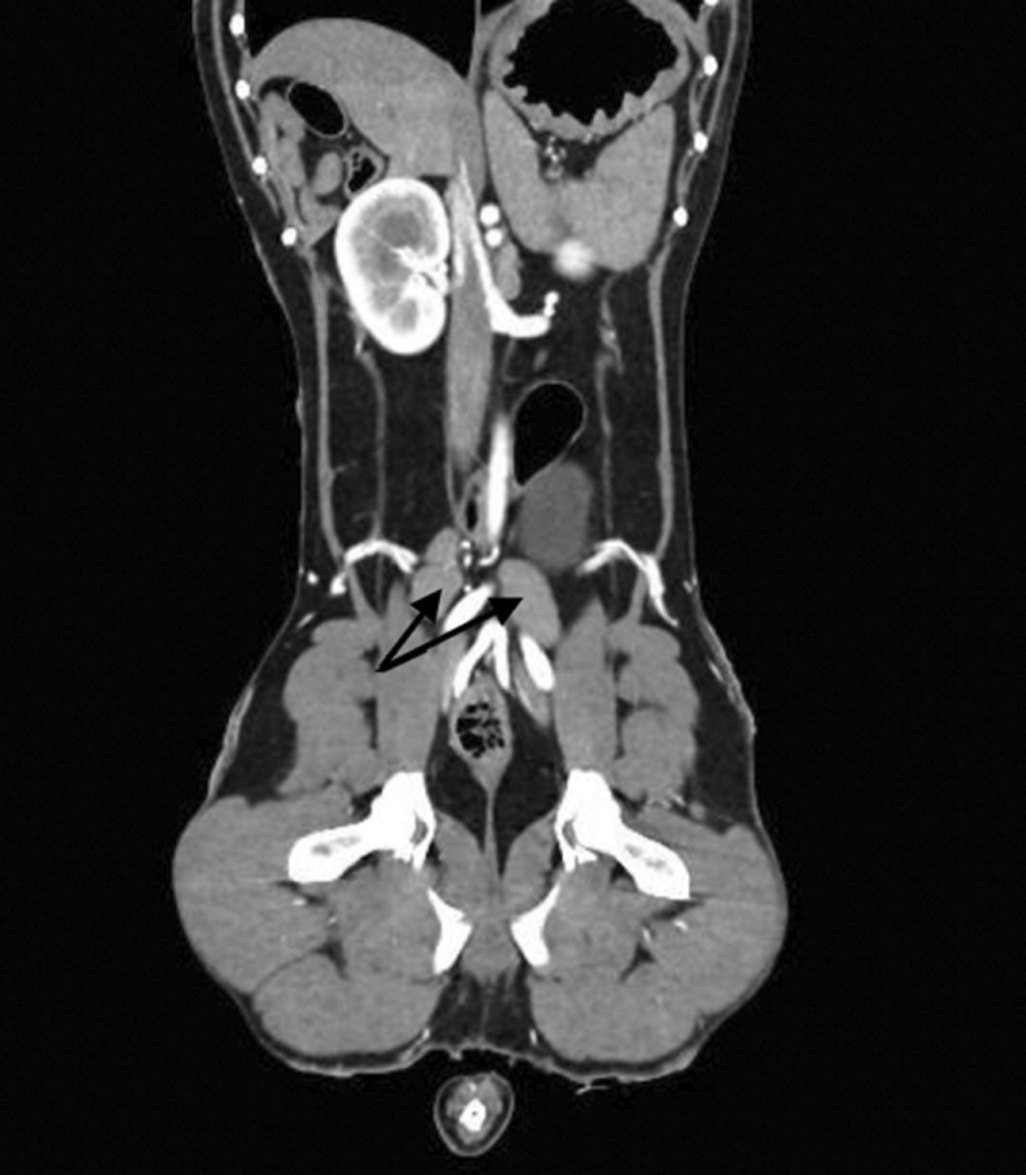
Post contrast dorsal plane CT image in soft tissue algorithm and window (WW400, WL40) demonstrating bilateral medial iliac lymphadenomegaly (black arrows).

Forty dogs underwent CT as part of their investigations, with lymphadenomegaly identified in 32 of 40 (80%) dogs. Of these 32 dogs, 16 (50%) dogs had peripheral lymphadenomegaly, seven (21.8%) dogs had both intrathoracic and intra‐abdominal lymphadenomegaly, six (18.7%) and three dogs (9.3%) had only thoracic and abdominal lymphadenomegaly, respectively. The medial iliac lymph nodes were most commonly enlarged (10/32) (Fig 2). Five of 40 (12.5%) dogs had no thoracic or abdominal abnormalities detected on CT. A full list of thoracic and abdominal CT findings is available in Tables 1 and 2, respectively.

Overall, thoracic radiography and thoracic CT were normal in 30 of 71 dogs (42%), while abdominal imaging (ultrasound and CT) was considered normal in 11 of 71 (15.4%) dogs.

Lesions consistent with endocarditis were identified on echocardiography in four of 13 (30.7%) dogs, with an audible cardiac murmur present in two (50%) of these dogs.

Cytology results from fine‐needle aspirates of lymph nodes, liver and spleen were available for 40 dogs (56%), and the main findings are summarised in Table 3. Additional cytological results included a subcutaneous abscess in two dogs and one each of: a prostatic abscess and pyogranulomatous inflammation (lung nodule).

**Table 3:** Cytology results from samples collected via ultrasound‐guided fine needle aspirate of abnormal findings

	FNA lymph nodes (*n* = 27), *n* (%)	FNA spleen (*n* = 12), *n* (%)	FNA liver (*n* = 9), *n* (%)
Reactive hyperplasia	22 (81.4)	4 (33.3)	
Extramedullary haematopoiesis		8 (66.6)	1 (11.1)
Vacuolar hepatopathy			5 (55.5)
Lymphoplasmacytic inflammation			1 (11.1)
Plasma cell hyperplasia sinus histiocytosis	1 (3.7)		
Hepatocellular hyperplasia			1 (11.1)
Chronic inflammation (macrophages and plasma cells)	1 (3.7)		
Lymphoma	1 (3.7)		
Lymphadenitis	2 (7.4)		
Histiocytic sarcoma			1 (11.1)

*n* Number of dogs

### Diagnostic utility of thoracic and abdominal imaging

Based on signalment, clinical signs, physical examination findings and clinicopathological results, 41 of 71 dogs were considered safe to immunosuppress without performing diagnostic imaging. Of these, 10 (24.4%) had abnormal diagnostic imaging findings suggestive of an underlying cause for their disease (secondary IMPA), thereby changing the clinicians' decision to proceed with immunosuppression. These included: endocarditis (2/10), suspected endocarditis (1/10), subcutaneous abscess (2/10), heterogeneous splenic mass and bilateral hypoenhancing renal nodules (1/10), generalised lymphadenomegaly (1/10), cavitary lung mass (1/10), hepatic target lesion and portosystemic shunt (1/10) and peritoneal effusion (1/10). The remaining 31 dogs had no abnormal imaging findings.

Thirty of 71 (42.2%) dogs would not have been immunosuppressed without first performing diagnostic imaging based on the above‐listed parameters. Of these, eight (26.6%) had diagnostic imaging findings cautioning against immunosuppression without further investigations. These included endocarditis (2/8), splenic mass (1/8), discospondylitis (1/8), hepatic mass (1/8), cellulitis and destruction of the right humeral head (1/8), prostatitis and a splenic nodule (1/8), myositis and sialadenitis (1/8), and multiple cystic lymph nodes, hypoechoic liver mass, heterogenous splenic lesion (1/8). In the remaining 22 of 30 (73.3%) dogs, diagnostic imaging was able to exclude an underlying trigger for their IMPA.

**FIG 3:**
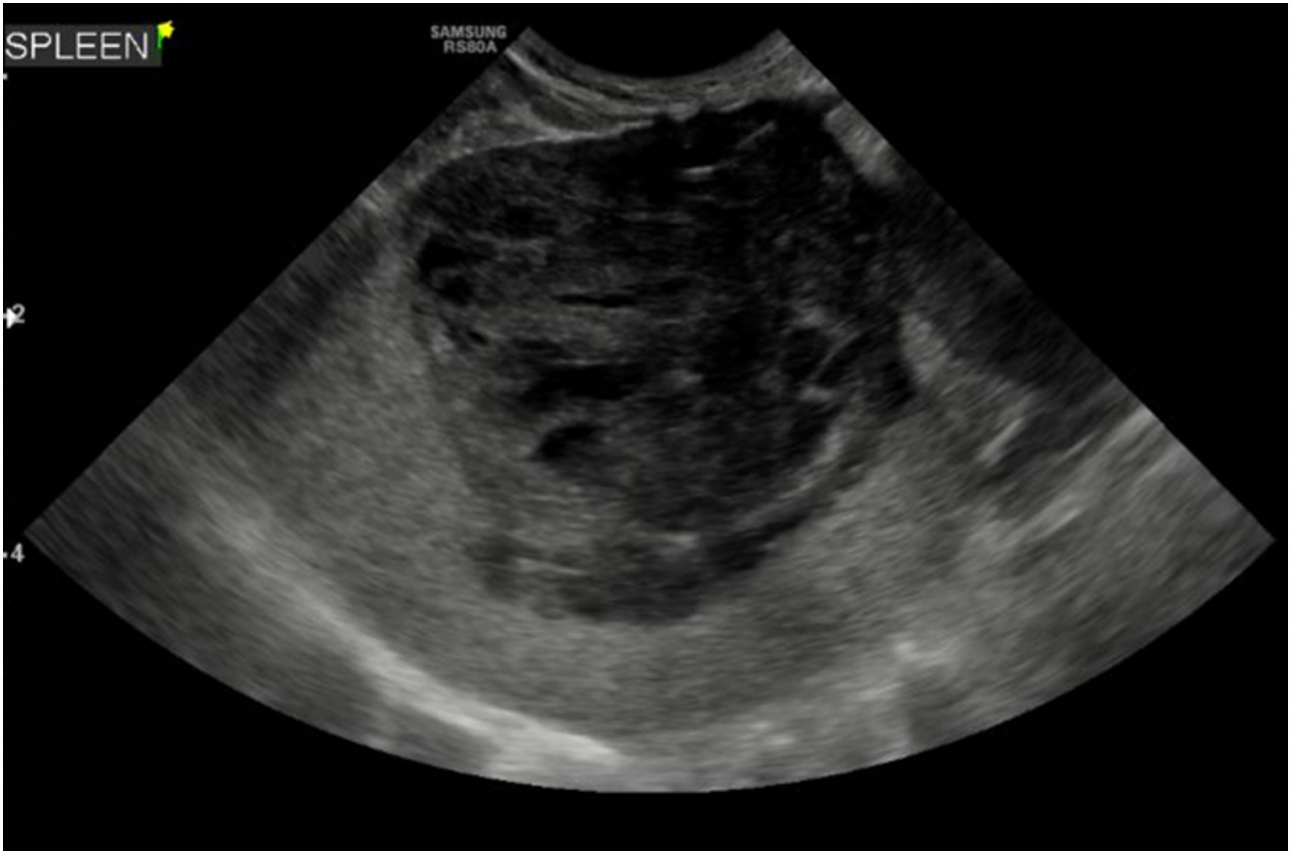
A large heterogeneous and cavitated splenic mass identified during abdominal ultrasound in a dog with IMPA, raising concern for an underlying neoplastic process. No neoplastic cells were identified on cytology of ultrasound‐guided aspirate of the lesion. Haematoma was histologically confirmed following splenectomy.

Overall, 18 of 71 (25.3%) dogs had diagnostic imaging findings suggestive of an underlying cause, and thus secondary IMPA. Following cytology neoplastic causes (Type IV) were confirmed in three dogs, while infectious triggers (Type II) were present in eight dogs, including endocarditis (four), subcutaneous abscess (two), prostatitis (one) and discospondylitis (one). Benign aetiology (Fig 3) was confirmed in five dogs via cytology or histology, while no sampling was performed in the remaining two (of 18) dogs. Further details on these dogs are included in Appendix S1.

## DISCUSSION

The primary aim of this retrospective study was to describe thoracic and abdominal imaging findings across modalities in dogs with IMPA.

Lymphadenomegaly was the most frequent imaging finding across CT and abdominal ultrasound studies, identified in 42 of 71 (59.1%) dogs. As previously reported (Ravicini et al., 2023), the medial iliac lymph nodes were most commonly affected. Lymphadenomegaly accompanies multiple immune‐mediated conditions reported in human and veterinary literature (De Laet et al., 2022; Ravicini et al., 2023; Ribas Latre et al., 2019; Schwartz et al., 2019). The lymphatic network is part of the immune system and therefore its involvement in IMPA is not surprising. However, it is important to recognise features of malignancy in enlarged lymph nodes of dogs with IMPA, because neoplasia is a possible underlying trigger for immune‐mediated disease (De Laet et al., 2022; Johnson & Mackin, 2012a). On ultrasound, round, hypoechoic or heterogeneous lymph nodes, are thought more likely to represent neoplastic infiltration (Kinns & Mai, 2007), although not all affected lymph nodes will exhibit such characteristics. Therefore, making the distinction between reactive and neoplastic lymph nodes requires cytological confirmation (Citi et al., 2020; Llabrés‐Díaz, 2004).

In this study, enlarged lymph nodes were commonly attributed to reactive hyperplasia at the time of image acquisition, and often not sampled. Where lymph nodes were sampled, reactive hyperplasia was the most common cytological diagnosis. While neoplasia was rare in this population, it was identified in three cases. The first of these had generalised lymphadenomegaly identified on CT, a final cytological diagnosis of lymphoma and, would have been immunosuppressed without performing diagnostic imaging based on the clinical signs and laboratory findings. The second dog would not have been immunosuppressed without performing diagnostic imaging based on the age and generalised lymphadenomegaly found on clinical examination. In this dog multiple heterogeneous cystic lymph nodes, as well as a hypoechoic liver mass and a heterogeneous splenic lesion were identified on abdominal ultrasound and cytology confirmed histiocytic sarcoma. The final case would not have been immunosuppressed without imaging based on the age as well as a new cardiac murmur. A large liver mass was identified on CT which was histologically confirmed as hepatocellular carcinoma. Echocardiography in this dog also confirmed endocarditis.

While multiple abnormalities were described in the imaging reports across the different modalities, only in 18 dogs (25.3%), were these considered significant enough to delay or avoid immunosuppression. The modalities that identified these abnormalities were CT (10/18), abdominal ultrasound (4/18) and echocardiography (4/18).

Thoracic radiography in our cohort of dogs revealed similar findings to recently published studies on immune‐mediated conditions, in which no significant findings were identified with this modality (Andres et al., 2019; De Laet et al., 2022; Tang et al., 2024). Thoracic radiography was completely normal in 58% of cases, with none of the described abnormalities in the remaining 42% of dogs having an impact on the final outcome of immunosuppression in these dogs. Similar to a recent publication (Tang et al., 2024), broncho‐interstitial (3/31) and interstitial (2/31) pulmonary patterns were most commonly described in this group of dogs, both of which are considered non‐specific findings. All thoracic radiographs were performed under sedation, therefore it is likely that in the absence of respiratory signs, these pulmonary patterns were a result of artefactual factors, such as poorly inflated lungs or body habitus (Spasov et al., 2018). An alveolar pattern was observed in one dog; however, this was not considered significant and attributed to atelectasis.

Immunosuppression is contraindicated in the presence of infections (Pressler et al., 2013). Therefore, ruling out an infectious focus is important in dogs with IMPA.

CT and echocardiography were the two modalities that most commonly identified septic foci in our cohort. Whilst echocardiography is not routinely performed in the initial investigations of IMPA, it identified lesions consistent with endocarditis in four dogs in our population, despite an audible cardiac murmur being present in only two of these (50%). This finding is in consensus with a recent study investigating infective endocarditis, where a cardiac murmur was present in 59% of cases, and suppurative inflammation was identified in 84% of dogs in whom arthrocentesis was performed (Sykes et al., 2006).

The main reason for performing diagnostic imaging in dogs with IMPA is to ensure they can be safely immunosuppressed and any possible causes of secondary IMPA have been ruled out. However, with the cost of veterinary care rising and clients likely to express financial concerns, it is important to know which investigations are likely to contribute towards decision making in dogs with IMPA while also allowing the clients to give truly informed consent. While both radiography and ultrasonography are readily available to most practices, CT is becoming increasingly used in the primary care setting. As demonstrated in this and other studies, thoracic radiography is unlikely to contribute to identifying an underlying cause in cases of IMPA (Andres et al., 2019; De Laet et al., 2022; Tang et al., 2024). Ultrasonography is highly operator dependent and performing a full diagnostic abdominal ultrasound can be challenging. On the other hand, acquisition of CT studies is comparably less skill‐dependent and presents a fast and non‐invasive way to assess abdominal structures. However, it is important to be aware that many findings identified on both ultrasonography and CT are non‐specific and therefore final diagnosis may rely on the clinician's ability to safely sample observed abnormalities.

Therefore, the secondary aim of this study was to assess the utility of diagnostic imaging in helping veterinarians decide which cases to immunosuppress and where diagnostic imaging should be prioritised.

Initially, the clinicians selected 41 of 71 dogs as safe to immunosuppress without the need for diagnostic imaging when presented with each dog's signalment, presenting signs, clinical examination and clinicopathological findings. This decision would have changed for ten dogs, following the review of diagnostic imaging findings. In one (of 10) case, the presence of a portosystemic shunt identified on CT alerted the clinicians to the possibility of bacteremia in this dog, prompting further investigations prior to immunosuppression (Meehan et al., 2016; Tinoco‐Najera et al., 2021). Although, the clinicians were able to correctly identify 31 dogs, which were indeed safe to immunosuppress, neoplasia, endocarditis and other infectious foci could have potentially been missed in the remaining ten dogs, had diagnostic imaging not been performed.

The remaining 30 of 71 dogs were considered likely to have secondary IMPA and, therefore, unsafe to immunosuppress without first performing diagnostic imaging. In 22 of these, diagnostic imaging was able to exclude an underlying cause for the IMPA. The eight dogs which were correctly identified by the internal medicine clinicians as likely to have secondary IMPA and in which abnormal findings were detected were older than 10 years of age or younger than 6 months of age, some had the presence of a cardiac murmur, and some had other visible signs such as a cutaneous swelling with a draining tract.

Of the total cohort, 18 (25.4%) dogs were suspected to be affected by secondary IMPA based on diagnostic imaging findings. These dogs were of varying age, with non‐specific presenting signs, including lethargy (12/18), pyrexia (8/18), pain (6/18), peripheral lymphadenomegaly (5/18), weakness (5/18), hyporexia/anorexia (4/18) and cardiac murmur (2/18). Following cytology of the imaging abnormalities, secondary IMPA was confirmed in 11 dogs, 15.5% of the total cohort.

Overall, the clinicians were able to correctly predict which dogs were safe to immunosuppress in 54.9% of the cases, based on the dog's signalment, presenting signs, clinical examination and clinicopathological results. The retrospective nature of this study has brought with it certain limitations.

Firstly, as the underlying causes that can trigger IMPA are often distant from the joints (Johnson & Mackin, 2012b; Ravicini et al., 2023), this study focused solely on thoracic and abdominal imaging. Therefore, the cases that only had musculoskeletal imaging performed were not included, thus reducing our population sample.

Secondly, the initial investigations performed varied based on the discipline that each dog presented to as well as the individual clinicians in charge of the case.

Thirdly, ultrasound is an inherently operator‐dependent modality, interpreted at the time of acquisition. Therefore, retrospective review of the images may not have represented the full extent of the findings outlined in the written reports accompanying the images.

Finally, this study has not looked at the outcomes of these dogs, nor included repeat imaging findings of the dogs that did not respond to initial treatment or who experienced relapses of their clinical signs. Therefore, future work with increased numbers of dogs, taking into consideration imaging findings of non‐responding or relapsing cases is needed to help us better understand this disease process and to better direct diagnostic work‐up.

In conclusion, abdominal imaging and echocardiography identified abnormal findings, suggestive of infectious or neoplastic aetiology, in a quarter of the cases in this study. Secondary IMPA was eventually confirmed in 15.5% of dogs following cytology. The clinicians were able to predict which dogs could be safely immunosuppressed without imaging in approximately half of the total cases. Therefore, our recommendation is to prioritise performing echocardiography and abdominal ultrasound or abdominal CT in dogs presenting with IMPA over other forms of imaging. Thoracic radiography may be selectively performed in cases when indicated by the presenting complaint or physical examination findings.

## Author contributions

**L. Atkinson:** Conceptualization (lead); data curation (lead); investigation (equal); methodology (equal); writing – original draft (lead); writing – review and editing (equal). **F. Schiborra:** Conceptualization (equal); data curation (equal); investigation (equal); methodology (equal); supervision (equal); writing – review and editing (equal). **E. O'Connell:** Conceptualization (equal); data curation (equal); investigation (equal); methodology (equal); supervision (equal); writing – review and editing (equal). **J. Barton:** Conceptualization (equal); data curation (equal); investigation (equal); methodology (equal); supervision (equal); writing – review and editing (equal). **W. Humphreys:** Conceptualization (equal); data curation (equal); investigation (equal); methodology (equal); supervision (lead); writing – review and editing (equal).

## Conflict of interest

None of the authors of this article has a financial or personal relationship with other people or organisations that could inappropriately influence or bias the content of the paper.

## Supporting information


**Appendix S1.** Summary of pertinent case details where diagnostic imaging identified abnormalities.

## Data Availability

Raw data were generated at Small Animal Teaching Hospital, University of Liverpool, as part of the diagnostic investigations of each patient. Data supporting the findings of this study are available by the corresponding author upon request.
