# Supplementary material for: Thoracic and abdominal diagnostic imaging findings in dogs diagnosed with immune‐mediated polyarthritis: 71 cases (2011‐2023)
Source: J Small Anim Pract. 2024 Dec 26;66(4):272–9. doi: 10.1111/jsap.13818 (PMC12000706; doi:10.1111/jsap.13818)
Supplement: Supplementary file 1 — Appendix S1. Summary of pertinent case details where diagnostic imaging identified abnormalities. [file JSAP-66-272-s001.docx]

**Appendix A – Summary of Pertinent Case Details where diagnostic imaging identified abnormalities.**

|  | **Would you immunosuppress this patient based on diagnostic imaging?** | **Would you immunosuppress this patient without performing diagnostic imaging?** | **Age**  **(months)** | **Clinical signs** | **Imaging findings** | **Abnormal Laboratory findings**  **(Reference range)** | **Cytology/final diagnosis** | **IMPA Type** |
| --- | --- | --- | --- | --- | --- | --- | --- | --- |
| 1 | No | No | 123 | - Pain - Lethargy - Pyrexia | Splenic mass | None | Splenic haematoma | Primary (Type I)- Idiopathic |
| 2 | No | Yes | 7 | - Lethargy - Anorexia, - Pain - Lameness - Pyrexia | Peritoneal effusion | - CRP- 63.9 mg/L   (<10mg/L) | Not sampled | Unknown |
| 3 | No | No | 86 | - Reluctant to walk - Pelvic limb weakness - Caudal abdominal pain | Cavitatory prostatomegaly | None | Acute prostatitis vs prostatic abscess | Secondary  (Type II)- infection |
| 4 | No | No | 58 | - Lethargy - Cardiac murmur - Peripheral lymphadenomegaly | Endocarditis | - ALP- 191 IU/L   (0-100 IU/L)   - CRP- 129 mg/L   (<10 mg/L) | Endocarditis | Secondary  (Type II)-infection |
| 5 | No | Yes | 32 | - Lethargy - Pyrexia - Intermittent lameness | Endocarditis | - CRP -73.1 mg/L (<10 mg/L) | Endocarditis | Secondary  (Type II)-infection |
| 6 | No | No | 120 | - Lethargy - Weakness - Generalised lymphadenomegaly - Pain | - Multiple cystic lymph nodes - Hypoechoic liver mass - Heterogenous splenic mass | None | Histiocytic sarcoma | Secondary (Type IV) – neoplasia |
| 7 | No | No | 4 | - Pain - Pottery gait | - Discospondylitis - Metaphyseal osteopathy | - CRP- 58.2 mg/L(<10 mg/L) - Creatinine kinase- 393.7 μmol/L (20-225μmol/L) - Albumin- 20.82 g/L (26.3-38.2 g/L) | - CSF- monocytoiod cells and small lymphocytes - Discospondylitis (C4-5) and metaphyseal osteopathy | Secondary  (Type II)-infection |
| 8 | No | Yes | 116 | - Lameness - Pelvic limb weakness - Lethargy - Hyporexia - Vomiting - Peripheral lymphadenomegaly | - Axillary and superficial cervical lymphadenomegaly - Small extrahepatic left gastric to cranial phernic portosystemic shunt - Hepatomegaly - Multiple renal infarcts | - Urea- 17.24 mmol/L (3.1-10.1mmol/L) - Creatinine- 148.3μmol/L (44-133μmol/L) - ALP- 395.1 IU/L (0-100 IU/L) - CRP- 108.6 mg/L (<10 mg/L) - UPCR- 2.1 mg/dL (<0.2 mg/dL) | - Liver – vacuolar hepatopathy - Spleen- extramedullary haematopoiesis - Lymph node- reactive hyperplasia - Iris stage II chronic kidney disease | Primary (Type I)- Idiopathic |
| 9 | No | No | 122 | - Lameness - Cardiac murmur | - Hepatic mass - Endocarditis | - ALP- 668 IU/L (0-100 IU/L) - CRP 11.6 mg/L (<10 mg/L) | - Endocarditis - Liver- hepatocellular carcinoma | Secondary-  (Type II) infection and (Type IV) neoplasia |
| 10 | No | Yes | 77 | - Shifting lameness - Generalised pain - Lethargy - Crusty non-pruritic lesions over dorsum | Generalized lymphadenomegaly | - Neutrophils -16 10e^9^/L (3-12 10e^9^/L) - Albumin -19.7 g/L (26.3-38.2 g/L) - ALP- 153 IU/L (0-100 IU/L) - CRP- 221 mg/L (<10 mg/L) | - Lymph nodes- multicentric lymphoma - Skin- non-degenerate neutrophils and acantholytic keratinocytes (pemphigus foliaceus) | Secondary (Type IV) – neoplasia |
| 11 | No | Yes | 60 | - Lameness - Unable to stand/move without assistance | - Rim enhancing structure left biceps - Left axillary lymphadenomegaly - Splenic nodules | - CRP -11.1 mg/L   (<10 mg/L)   - CK- 534U/L (10-200U/L) | - Lymph node – reactive hyperplasia - Splenic nodules- reactive hyperplasia - Biceps – neutrophilic inflammation, abscess. | Secondary  (Type II)-infection |
| 12 | No | No | 3 | - Progressive lameness - Lethargy | - Right shoulder effusions and destruction of humeral head - Medial brachial cellulitis with fluid pockets - Right prescapular, axillary and sternal lymphadenomegaly | - Albumin- 23.4 g/L (26-38 g/L) | - Septic arthritis suspected -culture negative - Lymph nodes not sampled | Secondary  (Type II)-infection |
| 13 | No | Yes | 84 | - Progressive lethargy - Pyrexia - Shifting lameness - Coughing and retching when eating and drinking - Left popliteal and prescapular lymphadenomegaly | - Cavitatory left caudal lung lobe mass - Generalised lymphadenomegaly | - Neutrophils- 1910e^9^/L (3-1210e^9^/L) - CRP- 123 mg/L   (<10 mg/L) | - Lymph nodes- reactive hyperplasia - Lung mass- endogenous lipid pneumonia | Primary (Type I)- Idiopathic |
| 14 | No | Yes | unknown | - Low head carriage - Lameness - Pyrexia | - Subcutaneous abscess left axillary region - Left axillary, sternal and cranial mediastinal lymphadenomegaly | - CRP- 218.2 mg/L   (<10 mg/L) | - Abscess with heavy growth of staphylococcus pseudointermidius | Secondary  (Type II)-infection |
| 15 | No | Yes | 91 | - Collapse - Pyrexia - Lethargy - Weight loss - Bilateral purulent aural discharge - Lameness | - Heart base nodule (chemodectoma suspected) - Endocarditis | - Neutrophils- 38 10e^9^/L (3-1210e^9^/L) - CRP 39.2 mg/L   (<10 mg/L) | - Ears- septic neutrophilic inflammation - Endocarditis | Secondary  (Type II)-infection |
| 16 | No | Yes | 33 | - Ulcerated nose - Weight loss - Pyrexia - Lethargy - Anorexia - Polyuria and polydipsia | - Splenomegaly - Suspicious of endocarditis | - CRP- 217.6 mg/L - (<10 mg/L) - UPCR- 2.19 mg/dL (<0.2 mg/dL) | - Skin staphylococcus pseudointermedius pyoderma - Spleen- reactive hyperplasia - No endocarditis | Primary (Type I)- Idiopathic |
| 17 | No | No | 72 | - Ventral neck swelling with a discharging sinus - Hyporexia - Lameness - Weight loss - Pyrexia | - Fascial swelling with associated oedema and heterogenous enhancement - Mandibular osteomyelitis - Multiple lung nodules - Axillary lymphadenoemgaly - Caudal right mammary nodule - Splenic nodules | - Neutrophils -15 10e^9^/L (3-1210e^9^/L) - Globulins -52.7 g/L (23.4-42.3 g/L) - CRP- 116.4 mg/L   (<10 mg/L) | - Discharging tract – Streptococcus canis and Pasteurella canis - Liver and spleen- neutrophilia - Lung nodules- pyogranulomatous inflammation - Lymph nodes: pyogranulomatous inflammation | Secondary  (Type II)-infection |
| 18 | No | Yes | 39 | - Pyrexia - Lethargy - Hyporexia - Lameness - Peripheral lymphadenomegaly | - Splenic mass - Pulmonary nodule - Renal nodules | - CRP- 285.7 mg/L   (<10 mg/L) | Not sampled | Unknown |

Note: C-reactive protein (CRP), Alkaline phosphatase (ALP), Urine Protein Creatinine Ratio (UPCR), Creatinine kinase (CK).
